# Flux flow spin Hall effect in type-II superconductors with spin-splitting field

**DOI:** 10.1038/s41598-019-42034-y

**Published:** 2019-04-11

**Authors:** Artjom Vargunin, Mikhail Silaev

**Affiliations:** 1 Department of Physics and Nanoscience Center, University of Jyväskylä, Jyväskylä, P.O. Box 35 (YFL), FI-40014 Finland; 2 Institute of Physics, University of Tartu, Tartu, EE-50411 Estonia

## Abstract

We predict the very large spin Hall effect in type-II superconductors whose mechanism is drastically different from the previously known ones. We find that in the flux-flow regime the spin is transported by the spin-polarized Abrikosov vortices moving under the action of the Lorenz force in the direction perpendicular to the applied electric current. Due to the large vortex velocities the spin Hall angle can be of the order of unity in realistic systems based on the high-field superconductors, superconductor/ferromagnet hybrid structures or the recently developed superconductor/ferromagnetic insulator proximity structures. We propose the realization of high-frequency pure spin current generator based on the periodic structure of moving vortex lattices. We find the patterns of charge imbalance and spin accumulation generated by moving vortices, which can be used for the electrical detection of individual vortex motion. The new mechanism of inverse flux-flow spin Hall effect is found based on the driving force acting on the vortices in the presence of injected spin current which results in the generation of transverse voltage.

## Introduction

The spin Hall effect (SHE) is currently one of the basic tools in spintronics used for the generation and detection of pure spin currents^1^. Although it has quite a rich variety of applications, from the fundamental point of view there have been only two known mechanisms leading to the spin Hall effect: (i) the spin-orbital interaction in semiconductors and heavy metals and (ii) the Zeeman spin splitting in graphene close to the neutrality point making the electrons and holes to carry different spin polarizations^2–4^. Here we suggest the third fundamental mechanism combining the specific properties of the electronic spectrum in superconductors with spin-splitting field and the coherent dynamics of the superconducting order parameter manifested through the flux flow of Abrikosov vortices under the action of the external transport current.

The non-equilibrium properties of superconductors with spin-splitting fields have become a hot topic in the field of superconductivity^5^. Such systems are characterized by the spin-dependent electron-hole asymmetry of Bogolubov quasiparticles^6^. Recently it has been realized that this feature allows for the generation of long-range spin accumulation^5,7–11^, which is robust against the usual spin-flip and spin-orbital scattering relaxations. This mechanism explains many experimental observations of long-range non-local spin signals in mesoscopic superconducting wires generated by the injected current from the ferromagnetic or even non-ferromagnetic electrodes^12–15^. In this paper we demonstrate the possibility of not only the long-range spin accumulation but also the non-decaying pure spin current generation using the properties of superconductors with spin-splitting fields.

In principle, the paramagnetic spin-splitting of Bogolubov quasiparticles appears inevitably due to the Zeeman effect in any superconductor subject to the magnetic field^12,14,16,17^. However, the magnetic field simultaneously leads to the orbital effect, inducing the center-of mass motion of the Cooper pairs due to the Meissner effect. The relative magnitude of the paramagnetic shift and the orbital kinetic energy of the Cooper pair is determined by the parameter introduced by Maki^16^ (referred later as the Maki parameter) ${\alpha }_{0}={\mu }_{B}/(eD)$, where *μ*_*B*_ is the Bohr magneton, *D* is the diffusion coefficient, *e* is the electron charge and we use theoretical units $c=\hslash ={k}_{B}=1$. Usually the orbital effect in superconductors dominates over the paramagnetic one, provided that the second critical field *H*_*c*2_ is not too high so that ${\mu }_{B}{H}_{c2}\ll {T}_{c}$. In this case the Maki parameter is small ${\alpha }_{0}\ll 1$. Exceptions are the high-field superconductors were the Zeeman shift can become relatively large at fields not exceeding *H*_*c*2_^16,18–22^. The paramagnetic effect can be significantly enhanced due to the geometrical confinement in thin superconducting films^12,14,23,24^. Alternatively, the spin splitting in superconductors can be induced by the exchange interaction of conduction electrons with localized magnetic moments, e.g. aligned magnetic impurities^25^ or in superconductor/metallic ferromagnet hybrid structures^26–28^. Recently, the systems consisting of superconducting films grown on the surfaces of ferromagnetic insulators like EuS^13,29–32^ and GdN^33^ have been fabricated. The exchange field ***h***_*eff*_ in the superconducting film is induced due to the scattering of conductivity electrons from the ferromagnetic insulator interface^34^. Such systems are currently studied quite actively as the possible platforms for the advanced radiation sensing technology^5,35^ and quantum computing with Majorana states^36^.

**Figure 1:**
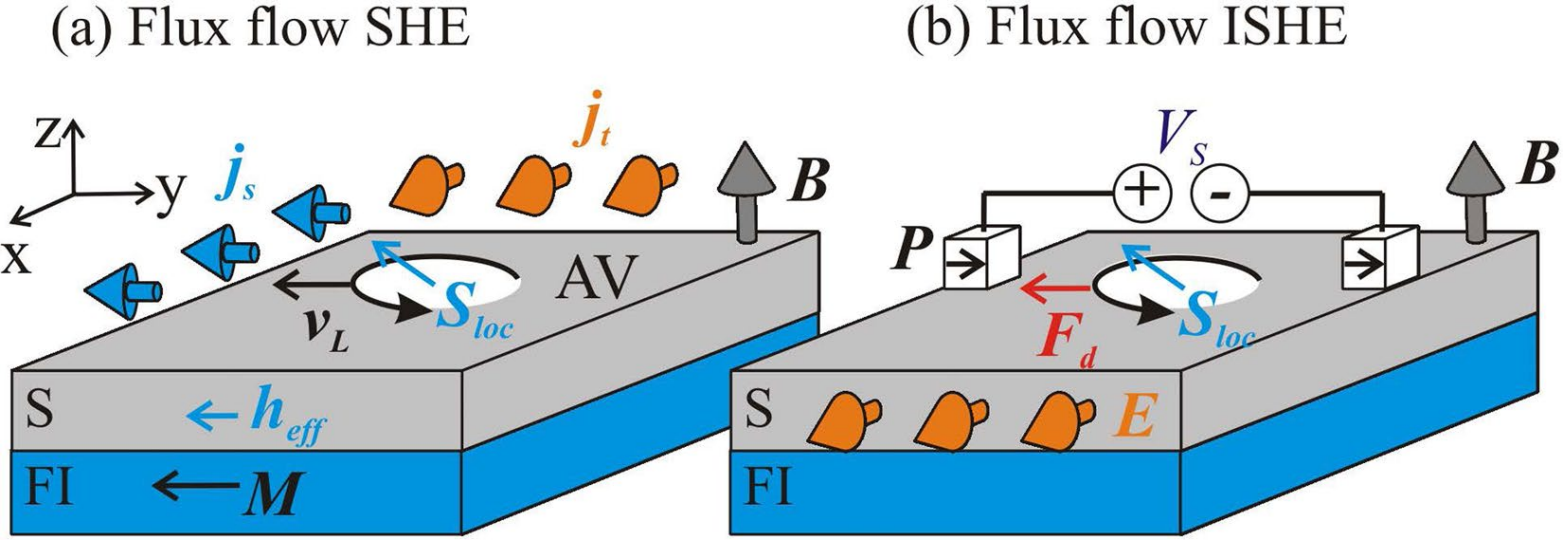
The mechanisms of direct and inverse flux-flow spin Hall effects. The schematic picture of (**a**) flux-flow spin Hall effect (SHE) and (**b**) flux-flow inverse spin Hall effect (ISHE) in type-II superconductors. Magnetic field ***B*** perpendicular to film plane creates Abrikosov vortices (AV). Vortex cores contain localized spin polarization ***S***_*loc*_ due to the splitting field ${\boldsymbol{h}}={{\boldsymbol{h}}}_{eff}+{\mu }_{B}{\boldsymbol{B}}$. (**a**) Transport current ${\boldsymbol{j}}={{\boldsymbol{j}}}_{t}$ generates the Lorentz force driving motion of spin-polarized AV with the velocity ${{\boldsymbol{v}}}_{L}\perp {\boldsymbol{j}}$ and inducing the spin current ${{\boldsymbol{j}}}_{s}\parallel {{\boldsymbol{v}}}_{L}$. (**b**) Ferromagnetic electrodes with polarization ***P*** generate spin-dependent bias *V*_*s*_. The induced spin accumulation gradient $\nabla {\mu }_{s}$ produces the driving force on AV, ${{\boldsymbol{F}}}_{d}\parallel \nabla {\mu }_{s}$ which results in the AV motion in the direction ${{\boldsymbol{v}}}_{L}\parallel \nabla {\mu }_{s}$ and induction of the average electric field ${\boldsymbol{E}}={\boldsymbol{B}}\times {{\boldsymbol{v}}}_{L}$.

The most well known paramagnetic effects in spin-singlet superconductors are the first-order transition into the normal state^18,19^ and the second-order transition into the inhomogeneous superconducting state induced by the spin-splitting field ***h***_*eff*_. The inhomogeneous state (FFLO) suggested by Fulde, Ferrell^25^ and Larkin, Ovchinnikov^37^ is realized in the narrow window of parameters and suppressed by impurities^38^ which hinders its experimental realizations^39^. However the first-order transition into the normal state driven by the Zeeman splitting has been detected in thin aluminum films^23^. In this paper we focus on the more robust nonequilibrium phenomena which generically appear in the presence of any spin-splitting field in the spin-singlet superconductor^5^. In particular, we consider the film of type-II superconductor which can host Abrikosov vortices. The example of such setup is shown schematically in Fig. 1. It consists of the thin superconducting film deposited on the magnetic insulator which creates spin splitting of the conduction electron subbands in the superconductor due to the effective exchange interaction ***h***_*eff*_. In addition there is a magnetic field ***B*** directed perpendicular to the film plane to create vortices. The total spin splitting field is given by the superposition ${\boldsymbol{h}}={\mu }_{B}{\boldsymbol{B}}+{{\boldsymbol{h}}}_{eff}$, so the single-particle Hamiltonian becomes $H={(i\nabla +e{\boldsymbol{A}})}^{2}/(2m)+\hat{{\boldsymbol{\sigma }}}{\boldsymbol{h}}$, where ***A*** is the vector potential and $\hat{{\boldsymbol{\sigma }}}$ is the vector of spin Pauli matrices.

Superconductors with the total spin splitting field ${\boldsymbol{h}}={{\boldsymbol{h}}}_{eff}+{\mu }_{B}{\boldsymbol{B}}$ coming both due to Zeeman shift and internal exchange are characterized by the renormalized Maki parameter $\alpha ={\alpha }_{0}h/({\mu }_{B}{H}_{c2})$. It can become large $\alpha \sim 1$ if the total spin splitting is close to the paramagnetic depairing threshold $h\sim {T}_{c}$. Such strong spin splitting has been recently obtained in superconductor/ferromagnetic insulator proximity structures used for the generation of the long-range spin accumulation in the non-local spin valve geometries^5,13,31–33^. Due to the large exchange field this regime can be achieved even if the Zeeman effect is small, that is when ${\mu }_{B}B\ll {T}_{c}$.

Although we focus on the superconductor/ferromagnetic insulator bilayer system, the regime when $\alpha \sim 1$ is also possible in high-field bulk superconductors where the spin splitting comes solely from the Zeeman effect^16,20^. Similar behavior can be observed in magnetic superconductors^40^, such as borocarbides^41^ where weak ferromagnetic ordering is possible^42^ so that vortex cores can host localized paramagnetic moments^43^ and weak pinning facilitates flux-flow regime. The effective spin-splitting can be obtained due to the inverse proximity effect in superconductor/metallic ferromagnet hybrids^26–28^. For instance, recently the flux-flow regime has been realized^44^ in Nb/PdNi/Nb trilayers which makes it potentially possible to study spin accumulation induced by vortex motion in such systems.

Below we demonstrate that *α* becomes the only relevant parameter which determines the amplitude of the pure spin current generated by the vortex motion. The latter can be characterized by the spin Hall angle ${\theta }_{sH}=e{j}_{s}/j$, where *j*_*s*_ is the induced spin current and and ${\boldsymbol{j}}={{\boldsymbol{j}}}_{t}$ is the charge current equal to the transport current generated by the external source. The spin Hall angle can be estimated as ${\theta }_{sH}\sim \alpha$. At the paramagnetic threshold $h\sim {T}_{c}$ it can reach ${\theta }_{sH}\sim 1$ which is much larger than the record values ${\theta }_{sH} < 0.1$ obtained in the heavy metal spin current generators^1^.

The above result is rather surprising because the maximal spin splitting $h\sim {T}_{c}$ is very small as compared to the Fermi energy ${\varepsilon }_{F}$, since in usual superconductors ${\varepsilon }_{F}/{T}_{c}\sim {10}^{2}-{10}^{3}$. In this case the polarization, which is the relative difference between spin-up/down conductivities is rather small ~$h/{\varepsilon }_{F}\ll 1$. This limit yields vanishing spin-polarized component of the resistive current. However, it is the vortex motion which generates much larger spin current in the transverse direction ${{\boldsymbol{j}}}_{s}\perp {\boldsymbol{j}}$. Large values of spin Hall angle are in the sharp contrast with extremely small ordinary (charge) Hall angle *θ*_*H*_. As shown by Kopnin^45^ the Hall angle is determined by the parameter ${\theta }_{H}\sim \tau {T}_{c}^{2}/{\varepsilon }_{F}$, where $\tau$ is the electronic mean free time due to the scattering by impurities. The first factor $\tau {T}_{c}\ll 1$ in the dirty regime while the second factor ${T}_{c}/{\varepsilon }_{F}\ll 1$ in all superconductors. So, ${\theta }_{H}\ll 1$ is determined by the product of two very small parameters, unlike the spin Hall angle which does not contain any small parameters. Thus, the charge and spin transport channels are separated in this system and the spin current generated by the vortex motion is to the very high accuracy the pure one with only vanishingly small admixture of the transverse charge current. Simultaneously, for ${\theta }_{H}\ll 1$ vortices move almost perpendicularly to the transport current ${{\boldsymbol{v}}}_{L}\perp {{\boldsymbol{j}}}_{t}$ according to the textbook picture of the flux-flow effect which we use in the discussion below.

The scheme of the flux-flow direct spin Hall effect is shown in Fig. 1a. Here, we assume that the superconductor with spin-splitting field and vortices is subject to the transport charge current ***j*** generated by the external source. This transport current induces the Lorenz force acting on the vortex lines in the direction perpendicular to current ${{\boldsymbol{F}}}_{L}\propto {\boldsymbol{j}}\times {\boldsymbol{B}}$. Provided that the Lorenz force overcomes the pinning barrier, vortices start to move in the transverse direction with the velocity ${{\boldsymbol{v}}}_{L}\perp {\boldsymbol{j}}$. Taking into account the spin polarization *S*_*loc*_ which exists inside each vortex core due to paramagnetic response, this motion generates the transverse pure spin current ${{\boldsymbol{j}}}_{s}\approx {n}_{v}{{\boldsymbol{v}}}_{L}{S}_{loc}$, where ${n}_{v}=B/{\varphi }_{0}$ is the vortex density, ${\varphi }_{0}$ is flux quantum.

Vortex cores in diffusive superconductors can be though of as the normal metal tubes, of the diameter determined by the coherence length $\xi$. In the presence of spin splitting field, the vortex cores contain localized spin ${S}_{loc}\sim {\chi }_{n}h{\xi }^{2}$ per unit vortex length, where ${\chi }_{n}={N}_{0}$ is the normal metal paramagnetic susceptibility and *N*_0_ is the Fermi-level density of states. To estimate *j*_*s*_ we substitute the flux-flow vortex velocity ${v}_{L}=-\,E/B$ and get ${\theta }_{sH}\sim h/(eD{H}_{c2})\sim \alpha$, so that *α* appears to be the only small parameter limiting the spin current generation. The physical reason for large *θ*_*sH*_ lies in the fast motion of vortices which can be compared e.g. with the Drude-model electron drift velocity $\bar{v}={\sigma }_{n}E/(ne)$, where the conductivity is ${\sigma }_{n}={e}^{2}{N}_{0}D$. At $B\approx {H}_{c2}$ we have the relation ${v}_{L}\approx \bar{v}{\varepsilon }_{F}/{T}_{c}\gg \bar{v}$. Therefore spin polarization can be transported much faster by moving vortices than by electrons drifting along the electric field.

Along with the direct SHE we propose also the scheme of the inverse flux-flow SHE shown in Fig. 1b. The mechanism is based on the injection of spin-polarized quasiparticle current into the superconductor by applying the voltage through the spin-filtering ferromagnetic electrodes with polarization ***P***. The resulting spin-dependent voltage *V*_*s*_ generates the spatially-inhomogeneous non-equilibrium spin accumulation which we hereafter denote *μ*_*z*_. Its gradient $\nabla$*μ*_*z*_ will be shown to produce the longitudinal force acting on the spin-polarized vortex cores pushing them towards one of the ferromagnetic electrodes. The vortex lattice motion with velocity ***v***_*L*_ generates electric field in the transverse direction ${\boldsymbol{E}}\parallel {\boldsymbol{B}}\times \nabla {\mu }_{s}$ thus providing the novel mechanism of inverse SHE.

## Model

To quantify these effects we use the framework of Keldysh-Usadel theory^46,47^ describing the spin current and spin accumulation induced by the vortex motion in the usual *s*-wave spin-singlet superconductor in the diffusive regime^5^. We consider the range of magnetic fields close to *H*_*c*2_, neglecting screening and using the Abrikosov solution for the moving vortex lattice. We will show that in addition to the large average spin current there is also the oscillating part which can be considered as the high-frequency source of the spin current at the nearly-terahertz range^48^.

We use the formalism of quasiclassical Green’s functions (GF)^46,47^ generalized to describe the non-equilibrium spin states in diffusive superconductors^5,49^, $$\breve{g}=(\begin{array}{cc}{\hat{g}}^{R} & {\hat{g}}^{K}\\ 0 & {\hat{g}}^{A}\end{array})$$, where ${\hat{g}}^{R/A/K}$ are the retarded/advanced/Keldysh components which are the matrices in spin-Nambu space and depend on two times and a single spatial coordinate variable $\breve{g}=\breve{g}({t}_{1},{t}_{2},{\boldsymbol{r}})$. Choosing the *z*-axis in spin space to be directed along the spin-splitting field ***h*** we consider general expressions for the spin density deviation from the normal state one, $S=S(t,{\boldsymbol{r}})$, and spin current ${\boldsymbol{j}}={{\boldsymbol{j}}}_{s}(t,{\boldsymbol{r}})$ determined through the GF at coinciding coordinates ${t}_{1,2}=t$

1 $$S=-\,\frac{\pi {\chi }_{n}}{8}{\rm{Tr}}[{\hat{\tau }}_{3}{\hat{\sigma }}_{3}{\hat{g}}^{K}]$$

2 $${{\boldsymbol{j}}}_{s}=\frac{\pi {\sigma }_{n}}{8{e}^{2}}{\rm{Tr}}[{\hat{\sigma }}_{3}{(\hat{g}\circ {\hat{\partial }}_{{\boldsymbol{r}}}\hat{g})}^{K}]$$

Here after ${\hat{\sigma }}_{i}$, ${\hat{\tau }}_{i}$ are the Pauli matrices in spin and Nambu spaces, and the symbolic time-convolution operator is given by $(A\circ B)\,({t}_{1},{t}_{2})=\int \,dtA({t}_{1},t)B(t,{t}_{2})$, the covariant differential superoperator is defined by ${\hat{\partial }}_{{\boldsymbol{r}}}=\nabla -ie{[{\hat{\tau }}_{3}{\boldsymbol{A}}]}_{t}$ and the two-time commutator is defined as ${[X,g]}_{t}=X({t}_{1})g({t}_{1},{t}_{2})-g({t}_{1},{t}_{2})X({t}_{2})$, similarly for anticommutator ${\{,\}}_{t}$. The Keldysh GF is conveniently described using the parametrization ${\hat{g}}^{K}={\hat{g}}^{R}\circ \hat{f}-\hat{f}\circ {\hat{g}}^{A}$ which follows from the normalization condition $\breve{g}\circ \breve{g}=\delta ({t}_{1}-{t}_{2})$. Here $\hat{f}=\hat{f}({t}_{1},{t}_{2},{\boldsymbol{r}})$ is the generalized distribution function. For calculations we use mixed representation $\breve{g}({t}_{1},{t}_{2})={\int }_{-\infty }^{\infty }\,\breve{g}(\varepsilon ,t){e}^{-i\varepsilon ({t}_{1}-{t}_{2})}d\varepsilon /2\pi$, where $t=({t}_{1}+{t}_{2})/2$ is the ‘center of mass’ time.

In the flux-flow regime we assume that vortices move with the constant velocity ***v***_*L*_. In the zero-order approximation the distribution function is equilibrium $\hat{f}(\varepsilon )={f}_{0}(\varepsilon ){\hat{\tau }}_{0}\equiv \,\tanh \,[\varepsilon /(2T)]{\hat{\tau }}_{0}$. Similarly, the spectral functions have their equilibrium forms in the frame moving together with vortices ${\hat{g}}^{R/A}({\boldsymbol{r}})\approx {\hat{g}}_{0}^{R/A}({\boldsymbol{r}}-{{\boldsymbol{v}}}_{L}t)$. This approximation yields zero spin current which is absent in equilibrium spin-singlet superconductors. Thus, we need to consider corrections in the linear-response regime which is realized provided the vortex velocity ***v***_*L*_ is small enough to neglect Joule heating, pair breaking or vortex-core shrinking effects^50,51^. For this purpose we take into account first-order terms in the gradient expansion of time convolutions^52,53^ as well as the non-equilibrium corrections to the spectral functions ${\hat{g}}_{ne}^{R/A}$ and the distribution function ${\hat{f}}_{ne}=\hat{f}-{f}_{0}{\hat{\tau }}_{0}$.

The nonequilibrium GF is determined by the Keldysh-Usadel equation^46,47^ which should be solved together with the self-consistency equations. In general this problem is very complicated and has never been approached even numerically. However, the regime of high magnetic fields ${H}_{c2}-B\ll {H}_{c2}$ allows for significant simplifications based on the existence of the Abrikosov vortex lattice solution for the superconducting order parameter. In this case it is possible to find analytically nonequilibrium corrections to the spectral functions ${\hat{g}}^{R/A}$ and the components of distribution function $\hat{f}$. First of all, we employ the analytical expression for the order parameter distribution in the moving vortex lattice. Assuming the particular directions of vortex velocity ${{\boldsymbol{v}}}_{L}={v}_{L}{\boldsymbol{y}}$ and electric field ${\boldsymbol{E}}=E{\boldsymbol{x}}$ we choose the time-dependent vector potential in the form ${\boldsymbol{A}}=Bx{\boldsymbol{y}}-Et{\boldsymbol{x}}$. Then the order parameter is given by superposition of the first Landau-level nuclei ${\mathcal L} (x)=\exp (\,-\,{x}^{2}/2{L}_{H}^{2})$, so that ${\rm{\Delta }}={b}_{0}{e}^{-2ieEtx}\,{\sum }_{n}\,{C}_{n}{e}^{inp(y-{v}_{L}t)} {\mathcal L} (x-n{x}_{0})$. Here *b*_0_ is magnetic field-dependent amplitude derived in the Supplementary Material, ${x}_{0}=p{L}_{H}^{2}$ determines the distance between neighbour superconducting nuclei and ${L}_{H}=1/\sqrt{2e{H}_{c2}}$ is the magnetic length. For the triangular lattice ${C}_{n+1}={e}^{i{(-1)}^{n}\pi /4}$, $p{L}_{H}=\sqrt{\pi \sqrt{3}}$ and for the square one ${C}_{n}=1$, $p{L}_{H}=\sqrt{2\pi }$.

Second, we use the known solutions for the equilibrium spectral functions in the vortex lattice near the upper critical field^54^. Here we take into account the spin-splitting field by shifting the quasiparticle energies according to ${\varepsilon }_{\sigma }=\varepsilon -\sigma h$, where $\sigma =\pm$. Then the spin-up ${g}_{0+}^{R}$ and spin-down ${\hat{g}}_{0-}^{R}$ GFs are given by

3 $${\hat{g}}_{0\sigma }^{R}({\boldsymbol{r}},\varepsilon )=[1+\frac{|{\rm{\Delta }}{|}^{2}}{2{(iq+{\varepsilon }_{\sigma })}^{2}}]\,{\hat{\tau }}_{3}+\frac{i|{\rm{\Delta }}|{\hat{\tau }}_{2}{e}^{-i\phi {\hat{\tau }}_{3}}}{iq+{\varepsilon }_{\sigma }},$$

and ${\hat{g}}_{0}^{A}=-\,{\hat{\tau }}_{3}{\hat{g}}_{0}^{R\dagger }{\hat{\tau }}_{3}$ for the advanced GF. Here $q=e{H}_{c2}D$ and the order parameter is ${\rm{\Delta }}=|{\rm{\Delta }}|{e}^{i\phi }$. The total GF is given by ${\hat{g}}_{0}^{R}={\hat{\sigma }}_{0}({\hat{g}}_{0+}^{R}+{\hat{g}}_{0-}^{R})/2+{\hat{\sigma }}_{3}({\hat{g}}_{0+}^{R}-{\hat{g}}_{0-}^{R})/2$.

**Figure 2:**
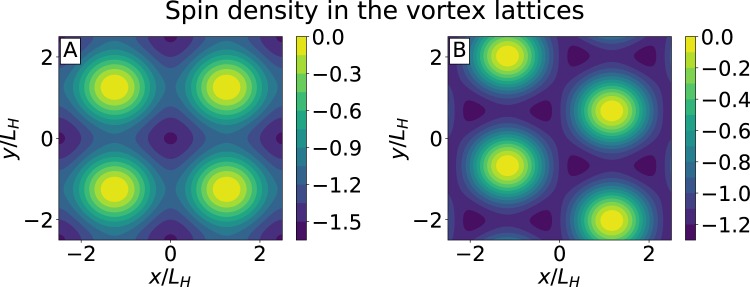
Spin density modulation in the vortex cores. Normalized deviation of the total spin density from the normal metal background, *S*/*S*_*n*_, on square (**A**) and triangular (**B**) lattices. Here ${S}_{n}=-\,{\chi }_{n}h$ is spin polarization of the normal metal and *S*/*S*_*n*_ is shown in the units of dimensionless order parameter amplitude $\langle {{\rm{\Delta }}}^{2}\rangle /{T}_{c}^{2}$. Calculations were performed at low-temperatures, $T\ll {T}_{c}$, for effective Maki parameter $\alpha =0.5$.

These spin-polarized spectral functions provide the description of equilibrium spin density modulation in a superconductor with spin-splitting field in the presence of vortex lattices. The periodic spin density patterns calculated for the typical cases of triangular and square lattices are shown in the Fig. 2. The spin polarization demonstrates enhancement at the vortex cores and suppression between vortices where the order parameter is larger. Thus even in the regime of dense vortex lattices there is an excess spin polarization *S*_*loc*_ localized in the vortex cores. It is natural to expect that the motion of such spin-polarized vortices will produce pure spin currents. Below we demonstrate the presence of these spin currents by an explicit calculation in the flux-flow regime considering the non-equilibrium situation when the vortex lattice moves under the action of the transport current ***j***_*t*_. We will calculate the spin current density induced by the vortex motion as well as the non-equilibrium spin accumulation and charge imbalance near the vortex cores.

## Results

### Spin current

The expression for spin current (2) can be decomposed into the parts ***j***_*s*1_ related to the distortions and time derivatives of spectral functions ${\hat{g}}^{R/A}$ and ***j***_*s*2_ which is determined by the corrections to the distribution function ${\hat{f}}_{ne}=\hat{f}-{f}_{0}$, where *f*_0_ is the equilibrium distribution function. The first part of the spin current ***j***_*s*1_ is determined by the non-equilibrium corrections to the spectral quantities while it contains only the equilibrium distribution function. In the charge sector these corrections yield the Caroli-Maki part of the flux-flow conductivity^55^. The important difference is that the charge current is determined by the corrections induced by the order parameter distortions in the moving vortex lattice while they do not contribute to the spin current. Since we neglect the spin-flip and spin-orbital scattering processes the total spin density and the spin current given by the Eqs (1) and (2) satisfy the continuity equation ${\partial }_{t}S+\nabla \cdot {{\boldsymbol{j}}}_{s}=0$. When implementing the linear response calculations we collect all contributions to the spin current to satisfy the continuity equation to the first order in vortex velocity. The derivation of this conservation law is discussed in the Supplementary Material.

The distortions of spectral GF generated by the vortex motion can be found using (3) and the normalization condition expanded in the mixed representation up to the first-order time derivatives. After performing several steps of analytical calculations described in Supplementary Material we obtain the spectral-related part of the spin current

4 $${{\boldsymbol{j}}}_{s1}=-\,\frac{{\sigma }_{n}}{16{e}^{2}}\frac{{\mathrm{Im}{\rm{\Psi }}}^{(2)}}{{(\pi T)}^{2}}{\rm{Re}}[{\rm{\Delta }}{(\hat{{\rm{\Pi }}}{\partial }_{t}{\rm{\Delta }})}^{\ast }-{\partial }_{t}{\rm{\Delta }}{(\hat{{\rm{\Pi }}}{\rm{\Delta }})}^{\ast }],$$

where ${\rm{\Psi }}={\rm{\Psi }}[1/2+(q+ih)/(2\pi T)]$ is digamma function, ${{\rm{\Psi }}}^{(n)}(z)={d}^{n}{\rm{\Psi }}(z)/d{z}^{n}$ and $\hat{{\rm{\Pi }}}=\nabla -2ie{\boldsymbol{A}}$. This part of the spin current has the non-zero space- and time-average $\langle {{\boldsymbol{j}}}_{s}\rangle =\langle {{\boldsymbol{j}}}_{s1}\rangle$. Indeed, as we show below the second part of the spin current related to the non-equilibrium distribution function does not contribute to the average $\langle {{\boldsymbol{j}}}_{s2}\rangle =0$.

At low temperatures, the space- average spin current can be written in the intuitively transparent expression

5 $$\langle {{\boldsymbol{j}}}_{s}\rangle =-\,{{\boldsymbol{v}}}_{L}\langle S\rangle (\frac{2}{1+{\alpha }^{2}})$$

where $\langle S\rangle$ is the average spin density deviation from the normal state. The absolute magnitude of $\langle S\rangle$ is determined by the average order parameter amplitude $\langle {{\rm{\Delta }}}^{2}\rangle$ which can be found analytically using the expression for the order parameter amplitude near the upper critical field^56,57^. Then, substituting $\langle S\rangle$ derived in the Supplementary Material and taking into account that the vortex velocity is ${v}_{L}=-\,j/({\sigma }_{n}{H}_{c2})$ we obtain the following analytical expression for the spin Hall angle ${\theta }_{sH}=e\langle {{\boldsymbol{j}}}_{s}\rangle /{{\boldsymbol{j}}}_{t}$, as a function of the average magnetic induction at low temperatures,

6 $${\theta }_{sH}(B)=-\,\frac{4\alpha }{{\beta }_{L}(1-{\alpha }^{4})}(1-\frac{B}{{H}_{c2}}),$$

where the Abrikosov parameter equals ${\beta }_{L}=1.16$ for the triangular and ${\beta }_{L}=1.18$ for the square lattice^58^. The growth of *θ*_*sH*_(*B*) with decreasing *B* given by Eq. (6) close to *H*_*c*2_ should continue at lower fields until the order parameter between vortices becomes fully developed at $B\approx 0.3{H}_{c2}$. In this regime ${\theta }_{sH}\propto \alpha$ without any small parameters so that ${\theta }_{sH}\sim 1$ for large exchange splitting $h\sim {T}_{c}$. Besides that, according to Eq. (6) large spin Hall angle can be obtained already in the regime $\mathrm{(1}-B/{H}_{c2})\ll 1$ provided that $1-\alpha \ll 1-B/{H}_{c2}$. Note that we restrict our consideration to $\alpha  < 1$ when the superconducting transition at $B={H}_{c2}$ is of the second order^20,59^.

Now let us consider the second part of the spin current determined by the correction to the distribution function. Due to the smallness of the order parameter near *H*_*c*2_ it can be written as ${{\boldsymbol{j}}}_{s2}=({\sigma }_{n}/{e}^{2})\nabla {\mu }_{s}$, where ${\mu }_{s}={\int }_{-\infty }^{\infty }\,{f}_{T3}(\varepsilon )d\varepsilon /2$ is the quantity which can be considered as the spin-dependent shift of the chemical potential and ${f}_{T3}={\rm{Tr}}[{\hat{\sigma }}_{3}\,\hat{f}]/4$ is the spin-dependent component of the distribution function^5,11^. Besides *f*_*T*3_ the vortex motion excites the electron-hole imbalance described by the component of distribution function ${f}_{T}={\rm{Tr}}[{\hat{\tau }}_{3}\,\hat{f}]/4$. Both these components are determined by the following kinetic equations (see Supplementary Material for derivation)

7 $$D{\nabla }^{2}{f}_{T3}=-\,{\partial }_{\varepsilon }\,{f}_{0}\{e{{\boldsymbol{J}}}_{se}{\boldsymbol{E}}+\frac{{\rm{Tr}}[{\hat{\sigma }}_{3}{\partial }_{t}\hat{{\rm{\Delta }}}({\hat{g}}_{0}^{R}-{\hat{g}}_{0}^{A})]}{8}\}$$

8 $$D{\nabla }^{2}{f}_{T}=-\,{\partial }_{\varepsilon }\,{f}_{0}\{e\nabla ({{\mathscr{D}}}_{T}{\boldsymbol{E}})-\frac{{\rm{Tr}}[{\hat{\tau }}_{3}{\partial }_{t}\hat{{\rm{\Delta }}}({\hat{g}}_{0}^{R}+{\hat{g}}_{0}^{A})]}{8}\},$$

where ${{\mathscr{D}}}_{T}=D{\rm{Tr}}({\hat{\tau }}_{0}{\hat{\sigma }}_{0}-{\hat{\tau }}_{3}\,{\hat{g}}_{0}^{R}{\hat{\tau }}_{3}\,{\hat{g}}_{0}^{A})/8$ is the diffusion coefficient for the charge imbalance modified by superconducting correlations, ${{\boldsymbol{J}}}_{se}=D{\rm{Tr}}[{\hat{\tau }}_{3}{\hat{\sigma }}_{3}({\hat{g}}_{0}^{R}\hat{\nabla }{\hat{g}}_{0}^{R}-{\hat{g}}_{0}^{A}\hat{\nabla }{\hat{g}}_{0}^{A})]/8$ is spectral spin-energy current density^11^ and the gap operator in the second term of the r.h.s. is $\hat{{\rm{\Delta }}}=i|{\rm{\Delta }}|{\hat{\tau }}_{2}{e}^{-i\phi {\hat{\tau }}_{3}}$. For the general-form Abrikosov vortex lattice, Eqs (7) and (8) can be solved analytically yielding the expression which can be found in the Supplementary Material. Since *f*_*T*3_ and hence *μ*_*s*_ have to be periodic functions, the contribution to spin current ***j***_*s*2_ has zero space and time-averages but contributes to the AC component of ***j***_*s*_.

**Figure 3:**
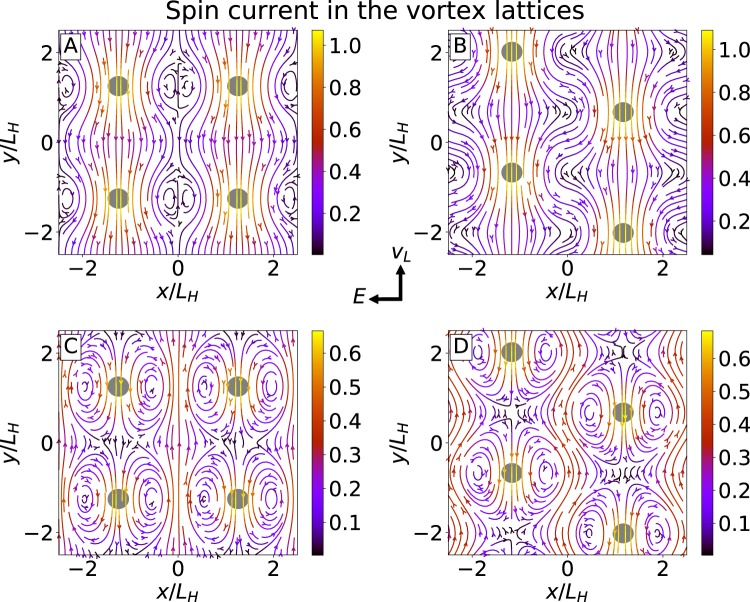
Spin current density generated by the vortex lattice motion. (**A**,**B**) The total spin current ***j***_*s*_ for square and triangular spin lattices generated by the vortex lattice motion, normalized by ${v}_{L}{\chi }_{n}\langle {{\rm{\Delta }}}^{2}\rangle /{T}_{c}$. (**C**,**D**) Deviation of the net spin current from its spatial average ${\tilde{{\boldsymbol{j}}}}_{s}={{\boldsymbol{j}}}_{s}-\langle {{\boldsymbol{j}}}_{s}\rangle$. Gray circles correspond to the position of the vortices. Left/right columns describe the case of the square/triangular lattices, respectively. Arrows between panels indicate the direction of the vortex velocity ***v***_*L*_ and average electric field ***E***. Calculations were performed at low-temperatures, $T\ll {T}_{c}$, for $\alpha =0.5$.

The overall distributions spin currents are shown in Fig. 3 produced using the Matplotlib package^60^ for two different vortex lattice geometries. Here one can see that the spin current mostly flows along the vortex chains with maximal current concentrated in the vortex cores. This result confirms our initial qualitative picture shown in Fig. 1 that the spin is transported by the moving spin-polarized vortex cores. In addition, in Fig. 3C,D one can see a non-trivial distribution of the spatially-periodic part of the current ${\tilde{j}}_{s}={{\boldsymbol{j}}}_{s}-\langle {{\boldsymbol{j}}}_{s}\rangle$, which is important for the AC spin current generation discussed below. The periodic part ${\tilde{{\boldsymbol{j}}}}_{s}$ forms two standing eddies localized close to the vortex core similar to that which are formed by the low-Reynolds viscous flow past a cylinder.

### Spin accumulation and charge imbalance

Besides generating the spin current, moving vortices produce other types of non-equilibrium states in the superconductor, such as the charge imbalance and the non-equilibrium spin accumulation which we denote as $\tilde{\mu }$ and ${\tilde{\mu }}_{s}$, respectively. These quantities have been widely used as the experimentally observable characteristics of the non-equilibrium superconducting states both in spin -degenerate^61–68^ and spin-split systems^12–15,24,31,69^. General expressions for charge imbalance and spin accumulation in terms of the quasiclassical GF read as

9 $$\tilde{\mu }=-\,\frac{\pi }{8}{\rm{Tr}}{\hat{g}}_{ne}^{K}(t,t,{\boldsymbol{r}})$$

10 $${\tilde{\mu }}_{s}=-\,\frac{\pi }{8}{\rm{Tr}}[{\hat{\tau }}_{3}{\hat{\sigma }}_{h}\,{\hat{g}}_{ne}^{K}]\,(t,t,{\boldsymbol{r}})$$

where ${\hat{g}}_{ne}^{K}$ is non-equilibrium part of Keldysh GF.

**Figure 4:**
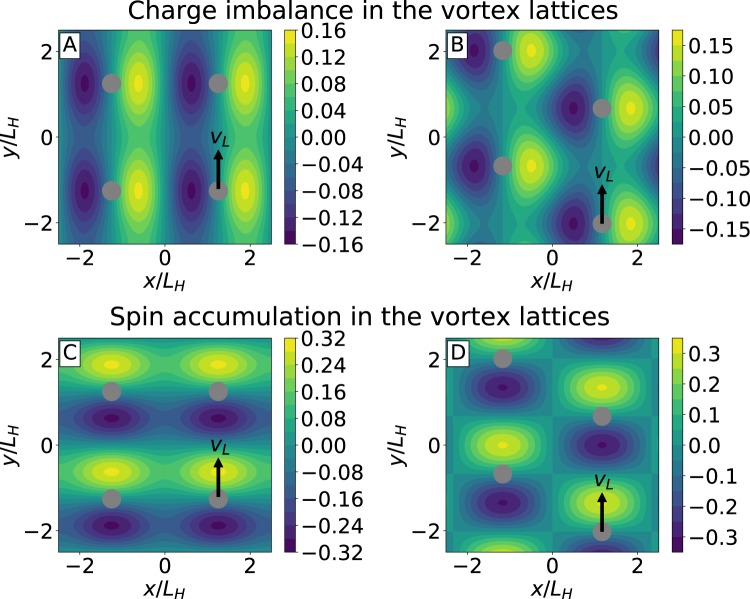
Charge imbalance $\tilde{\mu }$ (**A**,**B**) and spin accumulation ${\mathop{\mu }\limits^{ \sim }}_{s}$ (**C**,**D**) generated by the moving vortex lattices. Both quantities are normalized to ${v}_{L}\langle {{\rm{\Delta }}}^{2}\rangle /({L}_{H}{q}^{2})$. Left/right columns describe the case of the square/triangular lattices, respectively. Gray circles correspond to the position of the vortex cores and black arrows indicate the direction of the vortex velocity ***v***_*L*_. Calculations were performed at low-temperatures, $T\ll {T}_{c}$, for $\alpha =0.5$.

In contrast to the stationary cases considered before^5,49^ in the time-dependent system these imbalances are determined not only by the the nonequlibrium distribution function components $\mu =\int \,{f}_{T}d\varepsilon /2$ and *μ*_*s*_ discussed above. Besides that they contain additional terms determined by the dynamics of the order parameter

11 $${\tilde{\mu }}_{s}=-\,{\mu }_{s}+{v}_{L}{\partial }_{y}|{\rm{\Delta }}{|}^{2}\frac{{\mathrm{Im}{\rm{\Psi }}}^{(2)}}{{(4\pi T)}^{2}}$$

12 $$\tilde{\mu }=-\,\mu -{v}_{L}{\partial }_{x}|{\rm{\Delta }}{|}^{2}\frac{{\mathrm{Re}{\rm{\Psi }}}^{(2)}}{2{(4\pi T)}^{2}},$$

see the Supplementary Material for details of the derivation. Distributions of $\tilde{\mu }$ and ${\tilde{\mu }}_{s}$ generated by the moving triangular and square vortex lattices are shown in Fig. 4. The patterns of charge imbalance agree with the qualitative picture suggested by Bardeen and Stephen^70^ where the vortex motion is accompanied by the generation of dipolar-like electric field near the vortex core, corresponding to the electric dipole directed perpendicular to the vortex velocity ***v***_*L*_. On the contrary, the “spin dipoles” corresponding to the patterns of ${\tilde{\mu }}_{s}$ are directed along ***v***_*L*_. Note also, that spin accumulation is proportional to the generalized Maki parameter, while $\tilde{\mu }$ remains finite when $\alpha \to 0$. These quantities $\tilde{\mu }$ and ${\tilde{\mu }}_{s}$ can be directly measured with the help of ferromagnetic detector electrodes^5,12–15,24,31,62,65,67–69^ thus providing experimental tool to detect the motion of individual vortices.

### Flux-flow inverse spin Hall effect (ISHE)

We suggest the new mechanism of the flux-flow ISHE which is based on the previously unknown effect of longitudinal vortex motion driven by the spin current or spin accumulation injected into the superconductor from the attached ferromagnetic electrodes with polarization ***P***. We denote *V*_*s*_ the corresponding spin-dependent external bias. For simplicity we assume that the polarization is aligned with the spin-splitting filed in the superconductor ${\boldsymbol{P}}\parallel {\boldsymbol{h}}$. To calculate the force acting on vortex from the injected spin current we consider the regime of temperatures close to the critical one *T*_*c*_. In this case we can neglect the superconducting corrections to the density of states. This assumption simplifies expression for spin-dependent part of the distribution function which can be taken in the form corresponding to the normal metal ${f}_{T3}({\boldsymbol{r}})=-\,{\mu }_{s}({\boldsymbol{r}}){\partial }_{\varepsilon }\,{f}_{0}$ where ${\mu }_{s}={\mu }_{s}({\boldsymbol{r}})$ is the spatially-inhomogeneous spin accumulation generated by the external bias *V*_*s*_. Besides that here we consider the regime of small fields $B\ll {H}_{c2}$ when vortices can be considered as individual objects. The force acting on the single vortex from non-equilibrium spin-polarized environment ***F***_*d*_ can be calculated using the known general expression^52,53^. Near the critical temperature when $|{\rm{\Delta }}|\ll T$ we obtain the simple analytical result ${{\boldsymbol{F}}}_{d}\approx \nabla {\mu }_{s}{S}_{loc}|{\rm{\Delta }}{|}^{2}/{T}_{c}^{2}$, where *S*_*loc*_ is the total spin localized in the vortex core. This driving force, balanced by the friction ${{\boldsymbol{F}}}_{v}=-\,\rho {{\boldsymbol{v}}}_{L}$, where $\rho$ is the vortex viscosity coefficient, yields the flux-flow velocity ${{\boldsymbol{v}}}_{L}\parallel \nabla {\mu }_{z}$. Its absolute value can be found using the known analytical expression for viscosity coefficient $\rho ={\varphi }_{0}{\sigma }_{n}\beta {H}_{c2}$. The temperature dependence close to *T*_*c*_ is determined by the coefficient $\beta ={\beta }_{0}/\sqrt{1-T/{T}_{c}}$, where *β*_0_ is some numerical value^71–73^. Taking into account that the concentration of vortices is determined by the average magnetic induction *B* and using the usual expression for the sample-average electric field ${\boldsymbol{E}}=-\,{{\boldsymbol{v}}}_{L}\times {\boldsymbol{B}}$ we obtain the relation

13 $$\frac{eE}{\nabla {\mu }_{s}}\approx \frac{h}{q}\frac{|{\rm{\Delta }}{|}^{2}}{{T}_{c}^{2}}\frac{B}{{H}_{c2}}\sqrt{1-\frac{T}{{T}_{c}}}.$$

The obtained result (13) yields the linear response relation for the inverse spin Hall effect because the electric field *E* and the corresponding electric current are generated in response to the applied spin-dependent voltage *V*_*s*_. The overall temperature dependence of the generated electric field $E\propto {(1-T/{T}_{c})}^{3/2}$ is determined by the order parameter amplitude ${{\rm{\Delta }}}^{2}\propto (1-T/{T}_{c})$ and the additional factor which comes from the divergence of vortex viscosity coefficient close to the critical temperature^72^ $\rho \propto 1/\sqrt{1-T/{T}_{c}}$.

## Discussion and Conclusions

We have found the spin current generation by moving vortices which penetrate the whole volume of the type-II superconductor. Thus the obtained spin current in contrast to the previously known schemes based on the injection mechanisms exists everywhere in the sample volume and is prone to the spin relaxation mechanisms such as the spin-flip scattering. The predicted spin current generation can be tested in the open circuit geometries when the vortices annihilate at the insulating boundary. In this case the net spin current at the boundary, $y=0$, should vanish ${j}_{sy}(y=0)=0$ generating the surface spin accumulation ${V}_{s}={\mu }_{s}/e$ which can be measured by the ferromagnetic detector electrodes^12–15^.

The second possible experimental test is based on the direct measurement of the spin current injected through superconductor interfaces into the inverse spin Hall detector^74,75^. This approach allows for the measurement of both the DC and the high-frequency AC spin current signals. The latter is generated due to the periodic structure of moving vortex lattice. The distribution of the space-periodic spin current component is shown in Fig. 3C,D. The amplitude of AC component flowing through the superconductor interface, ${\langle {\tilde{j}}_{sy}\rangle }_{x}$, is determined by the variations of the current average along the boundary, ${\langle {j}_{sy}\rangle }_{x}$, with respect to the constant background current $\langle {j}_{s}\rangle$. At low temperatures, the relative magnitude is given by ${\langle {\tilde{j}}_{sy}\rangle }_{x}/\langle {j}_{s}\rangle =(1-{\langle {{\rm{\Delta }}}^{2}\rangle }_{x}/\langle {{\rm{\Delta }}}^{2}\rangle )\,(1+{\alpha }^{2})/2$. According to the recent measurements in Pb, the frequency of vortex entry into the superconducting sample can reach dozens of gigahertz^48^ and the THz range in layered high-temperature superconductors^76^. In the suggested setup this is the frequency of the AC spin current generated by the vortex motion. The high-frequency spin current generation can be useful in antiferromagnetic spintronics characterized by the terahertz-range dynamics of the magnetic system^77^.

The charge imbalance and spin accumulation have been accessed experimentally using non-local conductance measurements^12–15,24,31,62,65,67–69^, when the non-equilibrium states were created by the current in the injector circuit. The non-local electric signal has been measured between the normal detector electrodes, either ferromagnetic or non-ferromagnetic attached to the different points of superconducting sample. Here we show that in the flux-flow regime the non-equilibrium states with non-zero charge imbalance $\tilde{\mu }$ and spin accumulation ${\tilde{\mu }}_{s}$ appear in the absence of quasiparticle injector current, but rather just due to the vortex motion. The quantities $\tilde{\mu }$ and ${\tilde{\mu }}_{s}$ can be measured using the same electrical detection circuits as in the non-local conductance measurement setups. For example, the tunneling current at the non-ferromagnetic normal detector electrode is proportional to $\tilde{\mu }$. In case of the ferromagnetic electrode there is a contribution to the detector current^5^ proportional to ${\tilde{\mu }}_{s}$. In the flux-flow regime each vortex carries the distributions of $\tilde{\mu }$ and ${\tilde{\mu }}_{s}$ localized in the vortex core. Thus, moving vortices passing close to the detector electrode are expected to generate pulses of the tunneling current or voltage, depending on the detection scheme. This provides a tool capable for detecting the motion of individual vortices. In contrast to the magnetometer techniques it does not have the frequency limitations^48^ and therefore can directly resolve the ultrafast vortex motion with the frequencies up to the dozens of gigahertz.

To conclude, we have demonstrated fundamental mechanisms of direct and inverse spin Hall effects due to the flux-flow of Abrikosov vortices in type-II superconductors with spin-splitting field. The spin splitting can be generated by the adjacent ferromagnetic insulator as shown in Fig. 1, by the Zeeman effect in the magnetic field applied in the plane of thin superconducting film or due to the inverse proximity effect in superconductor/ferromagnet hybrid structures^26–28^. The pure spin current carried by the fast vortices moving in the transverse direction is characterized by the large spin Hall angle which in general does not contain any small parameters. Besides that there is also an AC component which appears due to the periodic structure of the vortex lattice. The AC spin current has the same order of magnitude as the average one. This effect can be used for the generation of spin signals in wide frequency domain up to the range of therahertz. We pointed out the longitudinal driving force exerted on vortex by the injected spin current. The vortex motion generated by this force leads to the inverse spin Hall effect. This mechanism can be applied for flux-flow based detection of pure spin currents.

## Supplementary information

Supplementary Material

## Data Availability

No datasets were generated or analysed during the current study.
