# Supplementary material for: Flux flow spin Hall effect in type-II superconductors with spin-splitting field
Source: Sci Rep. 2019 Apr 11;9:5914. doi: 10.1038/s41598-019-42034-y (PMC6459863; doi:10.1038/s41598-019-42034-y)
Supplement: Supplementary file 1 — Supplementary Material [file 41598_2019_42034_MOESM1_ESM.pdf]

Flux flow spin Hall effect in type-II superconductors with spin-splitting field: Supplementary Information

Artjom Vargunin[1,2] **and Mikhail Silaev**[1,*]

[1]Department of Physics and Nanoscience Center, University of Jyväskylä, P.O. Box 35 (YFL), FI-40014 University of Jyväskylä, Finland
[2]Institute of Physics, University of Tartu, Tartu, EE-50411, Estonia
[*]mikesilaev@gmail.com

The quasiclassical GF $\check{g}$ introduced in main text as block matrix in Nambu-spin space obeys in dirty limit the Usadel equation

$$\{\hat{\tau}_3\partial_t, \check{g}\}_t = D\hat{\partial}_{\mathbf{r}}(\check{g}\circ\hat{\partial}_{\mathbf{r}}\check{g}) + [\hat{H},\check{g}]_t \tag{S1}$$

where D is the diffusion constant, $\hat{H} = -|\Delta|\hat{\sigma}_0\hat{\tau}_2 e^{-i\varphi\hat{\tau}_3} - ih\hat{\sigma}_3\hat{\tau}_3$. Here $\hat{\tau}_i$ and $\hat{\sigma}_i$ $(i = 0,1,2,3)$ are Pauli matrices in Nambu and spin space, h is exchange field, and φ is gap phase. The (anti)commutator, symbolic product and differential superoperator in Eq. (S1) are defined in main text. Note that we consider temporal gauge where the scalar potential is zero $\Phi = 0$ with and additional constraint that in equilibrium the vector potential is time-independent and satisfies $\nabla\cdot\boldsymbol{A} = 0$. We also take theoretical units $c = \hbar = k_B = 1$.

Derivation of Eq. (3)

The spectral properties defined by diagonal contributions to Eq. (S1) can be evaluated by employing first-order gradient expansion as described in main text. Close to the upper critical field, H_{c2}, we expand non-stationary GF $\hat{g}^R = \begin{pmatrix} g_1 & g_2 \\ -g_2^+ & -g_1 \end{pmatrix}$ in orders of $|\Delta|$ and obtain linear equations

$$D\Pi_-^2 g_2 + 2i[(\varepsilon - h\hat{\sigma}_3)g_2 - \Delta\hat{\sigma}_0] = 0, \qquad D\Pi_+^2 g_2^+ + 2i[(\varepsilon - h\hat{\sigma}_3)g_2^+ - \Delta^*\hat{\sigma}_0] = 0, \tag{S2}$$

where $\Pi_\pm = \nabla \pm 2ie\boldsymbol{A}$. The diagonal part of the GF can be found from the normalization condition $g_1^2 = \mathbb{1} + g_2 g_2^+$. We apply gauge $\boldsymbol{A} = \boldsymbol{y}H_{c2}x - \boldsymbol{x}Et$ and use Abrikosov vortex lattice solution $\Delta = b_0 e^{-2ieEtx}\sum_n C_n e^{inp(y-v_L t)}\mathscr{L}(x - nx_0)$, see notations in main text, to get

$$g_2 = \frac{(\varepsilon + iq)\hat{\sigma}_0 + h\hat{\sigma}_3}{(\varepsilon + iq)^2 - h^2}\Delta, \qquad g_2^+ = \frac{(\varepsilon + iq)\hat{\sigma}_0 + h\hat{\sigma}_3}{(\varepsilon + iq)^2 - h^2}\Delta^*, \tag{S3}$$

where $q = DeH_{c2}$. This solution can be rewritten for separate spin up or down band, see Eq. (3) in the main text, and $v_L = E = 0$ should be substituted to obtain stationary limit, $\hat{g}_0^R$.

Derivation of Eqs. (4), (5) and (6)

We assume that vortices move with the constant velocity $\boldsymbol{v}_L$ and consider corrections in the linear-response regime which are realized provided the vortex velocity $\boldsymbol{v}_L$ is small enough. For this purpose we take into account first-order terms in the gradient expansion of time convolutions as well as the non-equilibrium corrections to the spectral functions $\hat{g}_{ne}^{R/A}$ and the distribution function $\hat{f}_{ne} = \hat{f} - f_0\hat{\tau}_0$. As a result, we get the two parts of spin current $\boldsymbol{j}_s = \boldsymbol{j}_{s1} + \boldsymbol{j}_{s2}$ given by

$$\boldsymbol{j}_{s1,2} = \frac{\sigma_n}{16e^2}\int_{-\infty}^{\infty}\mathrm{Tr}[\hat{\sigma}_3\hat{\boldsymbol{J}}_{1,2}^K]d\varepsilon, \tag{S4}$$

$$\hat{\boldsymbol{J}}_1^K = f_0(\hat{\boldsymbol{J}}_{ne}^R - \hat{\boldsymbol{J}}_{ne}^A), \tag{S5}$$

$$\hat{\boldsymbol{J}}_{ne}^{R/A} = (\hat{g}^{R/A}\hat{\partial}_r\hat{g}^{R/A})_{ne} - \frac{i}{2}(\partial_t\hat{g}_0^{R/A}\partial_\varepsilon\hat{\nabla}\hat{g}_0^{R/A} - \partial_\varepsilon\hat{g}_0^{R/A}\partial_t\hat{\nabla}\hat{g}_0^{R/A}), \tag{S6}$$

$$\hat{\boldsymbol{J}}_2^K = \hat{\partial}_r\hat{f} - \hat{g}_0^R\hat{\partial}_r\hat{f}\hat{g}_0^A + \hat{\boldsymbol{J}}_0^R\hat{f}_{ne} - \hat{f}_{ne}\hat{\boldsymbol{J}}_0^A. \tag{S7}$$

The first term in the r.h.s. of (S6) incorporates corrections to the spectral GF $g_{ne}^{R/A}$ as well as the electric field term which appears from the expansion of the covariant differential operator $\hat{\partial}_r\hat{X} = \hat{\nabla}\hat{X} + e\boldsymbol{E}\{\hat{\tau}_3, \partial_\varepsilon\hat{X}\}/2$ and $\hat{\nabla}X = \nabla\hat{X} - ie\boldsymbol{A}[\hat{\tau}_3,\hat{X}]$. Here we use

the gauge with zero electric potential such that electric field is given by $\boldsymbol{E} = -\partial_t \boldsymbol{A}$. The second term in the r.h.s. of (S6) comes from the linear-order expansion of time convolution. It contains the equilibrium spectral GF in the moving frame $\hat{g}_0^{R/A}(\boldsymbol{r} - \boldsymbol{v}_L t)$. The second part of the spin current $\boldsymbol{j}_{s2}$ is determined by the non-equilibrium distribution function. In the general case the differential operator in the r.h.s. of (S7) contains correction from time convolution expansion so that $\hat{\partial}_r \hat{f} = \nabla \hat{f} + e\boldsymbol{E}\hat{\tau}_3 \partial_\varepsilon f_0$.

In order to calculate contribution to spin current due to spectral GF, $\boldsymbol{j}_{s1}$, one should carefully take into account vortex-motion driven distortions of spectral GF, i.e. corrections to spectral GF, $\hat{g}^R_{ne} \sim v_L$, linear in vortex velocity. By using ansatz $\hat{g}^R \approx \hat{g}^R_0 + \hat{g}^R_{ne}$ and normalization condition we get

$$\hat{g}^R_0 \hat{g}^R_{ne} + \hat{g}^R_{ne} \hat{g}^R_0 = \frac{i}{2}(\partial_t \hat{g}^R_0 \partial_\varepsilon \hat{g}^R_0 - \partial_\varepsilon \hat{g}^R_0 \partial_t \hat{g}^R_0). \tag{S8}$$

This relation is used for calculating the first contribution to Eq. (S6), namely $(\hat{g}^R \hat{\nabla} \hat{g}^R)_{ne} = \hat{g}^R_{ne} \hat{\nabla} \hat{g}^R_0 + \hat{g}^R_0 \hat{\nabla} \hat{g}^R_{ne}$ so that

$$\mathrm{Tr}[\hat{\sigma}_3 (\hat{g}^R \hat{\nabla} \hat{g}^R)_{ne}] = \frac{1}{2}\mathrm{Tr}[\hat{\sigma}_3 \hat{\nabla}(\hat{g}^R_{ne}\hat{g}^R_0 + \hat{g}^R_0 \hat{g}^R_{ne})] = \frac{i}{4}\mathrm{Tr}[\hat{\sigma}_3 \hat{\nabla}(\partial_t \hat{g}^R_0 \partial_\varepsilon \hat{g}^R_0 - \partial_\varepsilon \hat{g}^R_0 \partial_t \hat{g}^R_0)], \tag{S9}$$

where short notation $\hat{\nabla} ab = (\hat{\nabla} a)b + a(\hat{\nabla} b)$ is used. Therefore, the spectral current density defined in (5) reads as $\hat{\boldsymbol{J}}^R_{ne} = i(\partial_\varepsilon \hat{g}^R_0 \hat{\nabla} \partial_t \hat{g}^R_0 - \partial_t \hat{g}^R_0 \hat{\nabla} \partial_\varepsilon \hat{g}^R_0)/2$ up to the terms which are traced out later. By using (S3), the contribution from $\hat{\boldsymbol{J}}^K_1 = f_0(\hat{\boldsymbol{J}}^R_{ne} - \hat{\boldsymbol{J}}^A_{ne})$ can be written as

$$\mathrm{Tr}[\hat{\sigma}_3 \hat{\boldsymbol{J}}^K_1] = i f_0 \left[\varepsilon_-^{-3} - \varepsilon_+^{-3}\right] \boldsymbol{I}_\Delta/2 + cc, \tag{S10}$$

where $\varepsilon_\pm = \varepsilon \pm h + iq$, $\boldsymbol{I}_\Delta = \Delta(\Pi \partial_t \Delta)^* - \partial_t \Delta (\Pi \Delta)^* + cc$ and $\Pi = \nabla - 2ieH_{c2} x \boldsymbol{y}$. We transform integral of (S10) over energy into the sum over Matsubara frequencies $\omega_n = \pi T(2n+1)$ to obtain

$$\int_{-\infty}^{\infty} \mathrm{Tr}[\hat{\sigma}_3 \hat{\boldsymbol{J}}^K_1] d\varepsilon = -4\pi i T \boldsymbol{I}_\Delta \sum_{n=0}^{\infty} \left[\omega_{n+}^{-3} - \omega_{n-}^{-3}\right] = -\frac{\boldsymbol{I}_\Delta \mathrm{Im}\Psi^{(2)}}{2(\pi T)^2}, \tag{S11}$$

where $\omega_{n\pm} = \omega_n \pm ih + q$, $\Psi = \Psi\left(\frac{1}{2} + \frac{q+ih}{2\pi T}\right)$ is digamma function and $\Psi^{(n)}(z) = \partial_z^n \Psi(z)$. This results in the Eq. (4). To find the spatial average of (S11) we first notice that due to Abrikosov vortex-lattice solution the components of vector $\boldsymbol{I}_\Delta$ reads as

$$\boldsymbol{I}_{\Delta x} = \frac{i v_L}{L_H^4} |b_0|^2 \sum_{n,m} C_n C_m^* e^{ipy(n-m)} \mathscr{L}(x_n)\mathscr{L}(x_m)\left[x_m^2 - x_n^2\right], \qquad \boldsymbol{I}_{\Delta y} = \frac{v_L}{L_H^4}|b_0|^2 \sum_{n,m} C_n C_m^* e^{ipy(n-m)} \mathscr{L}(x_n)\mathscr{L}(x_m)\left[x_n + x_m\right]^2, \tag{S12}$$

where $x_n = x - n x_0$. Stationary vortex-lattice solution is periodic function in y-direction with period $L_y = 2\pi/p$ and in x-direction with period $L_x = \nu x_0$, if $C_n = C_{n+\nu}$. Here $\nu = 1$ produces square and $\nu = 2$ triangular vortex lattice. Spatial average over vortex lattice reduces to Gaussian integrals so that $\langle \boldsymbol{I}_\Delta \rangle = 2\frac{v_L}{L_H^2}\langle \Delta^2 \rangle \boldsymbol{y}$, where $\langle \Delta^2 \rangle = \sqrt{\pi}|b_0|^2 L_H / x_0$. Combining this result with (S11) we obtain

$$\langle \boldsymbol{j}_{s1} \rangle = -\boldsymbol{v}_L \sigma_n \frac{\hbar \langle \Delta^2 \rangle \mathrm{Im}\Psi^{(2)}}{(4\pi k_B T e L_H)^2}, \tag{S13}$$

At the same time the average spin density deviation from the normal state induced by the superconducting correlations reads as

$$\langle S \rangle = -\chi_n \langle \Delta^2 \rangle \mathrm{Im}\Psi^{(1)}/(4\pi T). \tag{S14}$$

Low-temperature limits of Eqs. (S13) and (S14) result in the relation (5) and

$$\langle S \rangle \overset{T\to 0}{\to} \frac{\alpha \chi_n \langle \Delta^2 \rangle}{2q(1+\alpha^2)}. \tag{S15}$$

The absolute magnitude of the spin current depends on the order parameter amplitude b_0 which is determined by the magnetic field. In the limit of large Ginzburg-Landau parameter we get the usual expression for the average gap function[1,2] with the only substitution $\Psi^{(k)} \to \mathrm{Re}\Psi^{(k)}$ to get into account the presence of spin splitting. In the limit of low temperatures $T \ll T_c$ the amplitude is given by the analytical expression

$$\langle \Delta^2 \rangle = 4q^2 \left(1 - \frac{B}{H_{c2}}\right) \frac{1+\alpha^2}{\beta_L (1-\alpha^2)} \tag{S16}$$

where β_L is the Abrikosov parameter. Substituting this expression into (S15) and then the obtained $\langle S \rangle$ into (5), we get the spin Hall angle expression (6) by taking into account that near the upper critical field $v_L = -j/(\sigma_n H_{c2})$.

Spin conservation

The Keldysh part of Eq. (S1) reads as

$$\{\hat{\tau}_3\partial_t, \check{g}^K\}_t = D\hat{\partial}_{\mathbf{r}}(\check{g}\circ\hat{\partial}_{\mathbf{r}}\check{g})^K + [\hat{H}, \check{g}^K]_t \tag{S17}$$

and its components in the mixed representation are given up to the terms linear in vortex velocity by

$$[H, \check{g}^K]_t = [\hat{H}, \check{g}^K] - \frac{i}{2}\{\partial_t\hat{H}, \partial_\varepsilon\hat{g}_0^K\}, \qquad \{\hat{\tau}_3\partial_t, \check{g}^K\}_t = -i\varepsilon[\hat{\tau}_3, \hat{g}_0^K] + \frac{1}{2}\{\hat{\tau}_3, \partial_t\hat{g}_0^K\}, \qquad \hat{\partial}_{\boldsymbol{r}} \to \hat{\nabla} + \frac{e}{2}\boldsymbol{E}\{\hat{\tau}_3, \partial_\varepsilon\}, \tag{S18}$$

where $\hat{\nabla} = \nabla - ie\boldsymbol{A}[\hat{\tau}_3,]$ and $\boldsymbol{E} = -\partial_t\boldsymbol{A}$ is electric field in temporal gauge. We substitute this into Eq. (S17), multiply it by $-N_0\hat{\tau}_0\hat{\sigma}_3/16$, take trace and integrate over energy to obtain

$$\partial_t S + \nabla\cdot\boldsymbol{j}_s = -\frac{N_0 De}{16}\boldsymbol{E}\int_{-\infty}^{\infty} d\varepsilon\partial_\varepsilon \mathrm{Tr}(\hat{\tau}_3\hat{\sigma}_3 f_0(\hat{\boldsymbol{J}}_0^R - \hat{\boldsymbol{J}}_0^A)) - \frac{N_0}{16}\int_{-\infty}^{\infty} d\varepsilon\partial_\varepsilon \mathrm{Tr}(\hat{\sigma}_3 f_0(g_0^R - g_0^A)\partial_t\hat{\Delta}), \tag{S19}$$

where $\hat{\Delta} = i|\Delta|\hat{\tau}_2 e^{-i\varphi\hat{\tau}_3}$ and $\hat{\boldsymbol{J}}_0^{R/A} = \hat{g}_0^{R/A}\hat{\nabla}\hat{g}_0^{R/A}$. Since at high energies GF approach the normal-metal ones, $\hat{g}_0^{R/A} = \pm\hat{\tau}_3$, the integrals in the r.h.s. of Eq. (S19) vanish so that in the absence of the scattering processes spin is conserved.

Derivation and solution of Eq. (7)

Non-diagonal part of Usadel Eq. (S1) provides us with kinetic equation for the distribution function $\hat{f}$ which parametrizes the Keldysh GF $\hat{g}^K = \hat{g}^R\circ\hat{f} - \hat{f}\circ\hat{g}^A$. By subtracting the spectral components of Eq.(S1) from Keldysh part one obtains the equation

$$\hat{g}^R\circ\{\tau_3\partial_t, \hat{f}\}_t - \{\tau_3\partial_t, \hat{f}\}_t\circ\hat{g}^A = D\hat{\partial}_{\mathbf{r}}(\hat{\partial}_{\mathbf{r}}\hat{f} - \hat{g}^R\circ\hat{\partial}_{\mathbf{r}}\hat{f}\circ\hat{g}^A) + D\hat{g}^R\circ\hat{\partial}_{\mathbf{r}}\hat{g}^R\circ\hat{\partial}_{\mathbf{r}}\hat{f} - D\hat{\partial}_{\mathbf{r}}\hat{f}\circ\hat{g}^A\circ\hat{\partial}_{\mathbf{r}}\hat{g}^A + \hat{g}^R\circ[\hat{H}, \hat{f}]_t - [\hat{H}, \hat{f}]_t\circ\hat{g}^A. \tag{S20}$$

Here we omitted collision integral. Next we introduce four components of distribution function, $\hat{f} = f_L + f_{T3}\hat{\sigma}_3 + (f_T + f_{L3}\hat{\sigma}_3)\hat{\tau}_3$, related to energy, charge and spin imbalances. We need only kinetic equation for f_{T3} which can be obtained by using first-order gradient expansion of Eq. (S20) multiplied by $\hat{\sigma}_3$ and traced

$$\nabla\cdot(\mathscr{D}_L\nabla f_{T3} + \mathscr{D}_{T3}\nabla f_L + \boldsymbol{J}_e f_{L3} + \boldsymbol{J}_{se} f_T) = -e\partial_\varepsilon f_0\boldsymbol{E}\cdot\boldsymbol{J}_{se} - \frac{1}{8}\partial_\varepsilon f_0 \mathrm{Tr}[\hat{\sigma}_h\partial_t\hat{\Delta}(\hat{g}_0^R - \hat{g}_0^A)], \tag{S21}$$

where energy dependent diffusion coefficients and the spectral charge currents are

$$\mathscr{D}_L = D\mathrm{Tr}(\hat{\sigma}_0\hat{\tau}_0 - \hat{g}_0^R\hat{g}_0^A)/8, \qquad \mathscr{D}_{T3} = -D\mathrm{Tr}(\hat{\tau}_0\hat{g}_0^R\hat{\sigma}_3\hat{g}_0^A)/8,$$
$$\boldsymbol{J}_e = D\mathrm{Tr}[\hat{\tau}_3(\hat{g}^R\hat{\nabla}\hat{g}^R - \hat{g}^A\hat{\nabla}\hat{g}^A)]/8, \qquad \boldsymbol{J}_{se} = D\mathrm{Tr}[\hat{\tau}_3(\hat{g}^R\hat{\nabla}\hat{g}^R\hat{\sigma}_3 - \hat{\sigma}_3\hat{g}^A\hat{\nabla}\hat{g}^A)]/8. \tag{S22}$$

Close to H_{c2}, kinetic equation (S21) reduces to (7). By using vortex-lattice solution, the terms in r.h.s of (7) reads as

$$\boldsymbol{E}\cdot\boldsymbol{J}_{se} = EI_x^-\left[\varepsilon_-^{-2} - \varepsilon_+^{-2}\right]/8 + cc, \qquad \mathrm{Tr}[\hat{\sigma}_3\partial_t\hat{\Delta}(\hat{g}_0^R - \hat{g}_0^A)] = -I_t^+\left[\varepsilon_-^{-1} - \varepsilon_+^{-1}\right] - cc, \tag{S23}$$

where $I_x^\pm = \Delta^*\partial_x\Delta \pm cc$ and $I_t^\pm = \Delta^*\partial_t\Delta \pm cc$. By integrating (7) over energy and introducing spin accumulation $\mu_s = \int_{-\infty}^{\infty} f_{T3}d\varepsilon/2$ we obtain close to H_{c2}

$$\nabla^2\mu_s = eEI_x^-\pi T\sum_{n=0}^{\infty}\left[\omega_{n+}^{-3} - \omega_{n-}^{-3}\right] - \frac{I_t^+\pi Ti}{2D}\sum_{n=0}^{\infty}\left[\omega_{n+}^{-2} - \omega_{n-}^{-2}\right] = -\frac{ieEI_x^-}{2(2\pi T)^2}\mathrm{Im}\Psi^{(2)} + \frac{I_t^+}{4D\pi T}\mathrm{Im}\Psi^{(1)}. \tag{S24}$$

To obtain r.h.s of first line in (S24) we integrated by parts and then transformed energy integral into Matsubara sum. By using vortex-lattice solution, the source term is determined by

$$I_x^\pm = |b_0|^2\sum_{n,m} C_n C_m^* e^{ipy(n-m)}\frac{-x_n \mp x_m}{L_H^2}\mathscr{L}(x_n)\mathscr{L}(x_m), \tag{S25}$$

and $I_t^\pm = -iv_L I_x^\mp$. Next we consider overlap between nearest Gaussians only, $n-m = \pm 1$. As a result, Eq. (S24) has the form

$$\nabla^2\mu_s = -\frac{p}{L_H^2}F_0 v_L\sum_n \sin(py + \phi_n)\mathscr{L}(x_n - x_0)\mathscr{L}(x_n), \qquad F_0 = \langle\Delta^2\rangle\frac{pL_H}{2\sqrt{\pi}}\left(\frac{\mathrm{Im}\Psi^{(2)}}{(2\pi k_B T)^2} - \frac{\mathrm{Im}\Psi^{(1)}}{2\pi qk_B T}\right), \tag{S26}$$

and $\phi_n = \arg(C_n^* C_{n+1})$. The solution of differential Eq. (S26) has the form $\mu_s = \frac{v_L}{2L_H^2}F_0\sum_n \sin(py + \phi_n)F(x_n)$, where F is given by

$$F(x) = \int_{-\infty}^{\infty} e^{-p|x-x'|}\mathscr{L}(x' - x_0)\mathscr{L}(x')dx'. \tag{S27}$$

Derivation of Eq. (11)

Non-equilibrium corrections to spectral GF are determined by (S8) which in the leading order in $|\Delta|$ can be written as $\hat{\tau}_3\hat{g}_{ne}^{R/A} + \hat{g}_{ne}^{R/A}\hat{\tau}_3 = \pm\frac{i}{2}(\partial_t\hat{g}_0^{R/A}\partial_\varepsilon\hat{g}_0^{R/A} - \partial_\varepsilon\hat{g}_0^{R/A}\partial_t\hat{g}_0^{R/A})$. This results in two conditions

$$\mathrm{Tr}\hat{g}_{ne}^{R/A} = \pm\frac{i}{4}\mathrm{Tr}([\hat{\tau}_3\hat{\sigma}_0, \partial_t\hat{g}_0^{R/A}]\partial_\varepsilon\hat{g}_0^{R/A}), \qquad \mathrm{Tr}[\hat{\tau}_3\hat{\sigma}_3\hat{g}_{ne}^{R/A}] = 0. \tag{S28}$$

By using first-order gradient expansion of Keldysh GF and conditions (S28) we obtain in the leading order

$$\mathrm{Tr}\hat{g}_{ne}^{K} = 8f_T + if_0 I_t^- \left[\varepsilon_-^{-3} + \varepsilon_+^{-3} + cc\right]/2, \qquad \mathrm{Tr}[\hat{\tau}_3\hat{\sigma}_3\hat{g}_{ne}^{K}] = 8f_{T3} - i\partial_\varepsilon f_0 I_t^+ \left[\varepsilon_-^{-2} - \varepsilon_+^{-2} - cc\right]/2. \tag{S29}$$

By performing integration over energy by parts and transforming integral to the summation over Matsubara frequencies, spin accumulation near H_{c2} becomes

$$\tilde{\mu}_s = -\mu_s - I_t^+ \pi T i \sum_{n>0}\left[\omega_{n+}^{-3} - \omega_{n-}^{-3}\right]/2. \tag{S30}$$

After summation this results in Eq. (11). By considering only overlap between nearest-neighbour Gaussians we obtain at low temperatures $T \to 0$ asymptotic expression

$$\tilde{\mu}_s = -\frac{\nu_L\langle\Delta^2\rangle}{2\sqrt{\pi}L_H(eDH_{c2})^2}\frac{\alpha}{(1+\alpha^2)^2}\sum_n \sin(py+\chi_n)\mathscr{H}(x_n), \qquad \mathscr{H}(x) = 2(pL_H)^2\mathscr{L}(x-x_0)\mathscr{L}(x) + p\left[1+(1+\alpha^2)/2\right]F(x). \tag{S31}$$

Derivation and solution of Eq. (8)

To calculate electrostatic potential defined by first line in (S29), we first solve kinetic equation for charge imbalance f_T. The equation can be obtained by using first-order gradient expansion of Eq. (S20) multiplied by $\hat{\tau}_3$ and traced

$$\nabla\cdot(\mathscr{D}_T\nabla f_T + \mathscr{D}_{L3}\nabla f_{L3}) + \boldsymbol{J}_e\cdot\nabla f_L + \boldsymbol{J}_{se}\cdot\nabla f_{T3} - \mathscr{R}_T f_T - \mathscr{R}_{L3}f_{L3} + e\partial_\varepsilon f_0\nabla(\boldsymbol{E}\mathscr{D}_T) = \frac{1}{8}\partial_\varepsilon f_0\mathrm{Tr}[\hat{\tau}_3\partial_t\hat{\Delta}(\hat{g}_0^R + \hat{g}_0^A)]. \tag{S32}$$

where

$$\mathscr{D}_T = D\mathrm{Tr}(\hat{\tau}_0\hat{\sigma}_0 - \hat{\tau}_3\hat{g}_0^R\hat{\tau}_3\hat{g}_0^A)/8, \qquad \mathscr{D}_{L3} = -D\mathrm{Tr}(\hat{\tau}_3\hat{g}_0^R\hat{\tau}_3\hat{\sigma}_h\hat{g}_0^A)/8, \qquad \mathscr{R}_T f_T + \mathscr{R}_{L3}f_{L3} = -\mathrm{Tr}\tau_3(\hat{g}_0^R[\hat{H},\hat{f}] - [\hat{H},\hat{f}]\hat{g}_0^A)/8. \tag{S21}$$

Close to H_{c2}, where order parameter is small, kinetic Eq reduces to Eq. (8) which can be written with the help of vortex-lattice solutions as

$$\nabla^2 f_T = -eE\partial_\varepsilon f_0 I_x^+\left[\varepsilon_-^{-2} + \varepsilon_+^{-2} + i(\varepsilon_-^{-1} + \varepsilon_+^{-1})/q + cc\right]/8 - \partial_\varepsilon f_0 I_t^-\left[\varepsilon_-^{-1} + \varepsilon_+^{-1} - cc\right]/(8D). \tag{S33}$$

We integrate r.h.s. of (S33) over energy by parts and transform integrals to Matsubara sum to get for $\mu = \int_{-\infty}^{\infty} f_T d\varepsilon/2$ an equation

$$\nabla^2\mu = -\frac{eE}{2(2\pi T)^2}\left[\mathrm{Re}\Psi^{(2)} + \frac{4\pi T}{q}\mathrm{Re}\Psi^{(1)}\right]I_x^+. \tag{S34}$$

Particular solution of this inhomogeneous equation can be written in the approximation of nearest-neighbour Gaussians as

$$\mu = \frac{eE\langle\Delta^2\rangle pL_H}{2\sqrt{\pi}(2\pi T)^2}\left[\mathrm{Re}\Psi''(z) + \frac{4\pi T}{q}\mathrm{Re}\Psi'(z)\right]\sum_n G(x_n, y), \tag{S35}$$

$$G(x,y) = \cos(py+\chi_n)\left[\int_x^\infty dx' e^{p(x-x')}\mathscr{L}(x'-x_0)\mathscr{L}(x') - \int_{-\infty}^x dx' e^{-p(x-x')}\mathscr{L}(x'-x_0)\mathscr{L}(x')\right] - L_H\frac{\sqrt{\pi}}{2}\mathrm{erf}\left(\frac{x}{L_H}\right).$$

Note that last term in G produces linear divergence modulated by weak oscillation due to summation over n in μ. However, linear function is homogeneous solution of Eq. (S34), so that linear divergence in (S35) can be removed by substracting linear homogeneous solution from G.

Derivation of Eq. (12)

By using (S29), full electrostatic potential near H_{c2} reads as

$$\tilde{\mu} = -\mu - \frac{i}{32}\int_{-\infty}^{\infty} f_0 I_t^- \left(\varepsilon_-^{-3} + \varepsilon_+^{-3} + cc\right) d\varepsilon. \tag{S36}$$

After transforming energy integral to Matsubara sum we obtain Eq. (12). By using (S35) and asymptotic expansions of digamma function, we obtain at low temperatures

$$\tilde{\mu} = -\frac{v_L\langle\Delta^2\rangle}{2\sqrt{\pi}L_H(eDH_{c2})^2}\frac{1}{(1+\alpha^2)^2}\left[-p(1+3\alpha^2)\sum_n G(x_n,y)/2 - (1-\alpha^2)\sqrt{\pi}L_H\partial_x|\Delta|^2/(4\langle\Delta^2\rangle)\right]. \tag{S37}$$

References

1. Caroli, C., Cyrot, M. & de Gennes, P. G. The magnetic behavior of dirty superconductors. *Solid State Commun.* **4**, 17–19 (1966).
2. Silaev, M. Magnetic behavior of dirty multiband superconductors near the upper critical field. *Phys. Rev. B* **93**, 214509 (2016).
